# Development of the item pool for the ‘WHO-ageism scale’: conceptualisation, item generation and content validity assessment

**DOI:** 10.1093/ageing/afad105

**Published:** 2023-10-30

**Authors:** Aja L Murray, Vânia de la Fuente-Núñez

**Affiliations:** Department of Psychology, University of Edinburgh, 7 George Square, Edinburgh EH8 9JZ, UK; Demographic Change & Healthy Ageing Unit, Department of Social Determinants of Health, Division of Healthier Populations, World Health Organization, 1211 Geneva, Switzerland

**Keywords:** ageism, scale development, content validity, older people, younger people, measurement

## Abstract

**Objectives:**

ageism harms individuals’ health and wellbeing and can be costly to societies. Reliable and valid measures that can quantify ageism are critical for achieving accurate data on its global prevalence, determinants and impacts, and to evaluate the effectiveness of interventions to reduce it. Ageism scales exist; however, none have been demonstrated to validly measure ageism in a manner consistent with consensus definitions of the concept (i.e. as manifested in all of stereotypes, prejudices and discrimination), whilst also quantifying ageism against all groups, from a target and perpetrator perspective, and across diverse country settings. Our objective was to develop an item pool to meet this need.

**Methods:**

we completed the conceptualisation, item generation and content validity assessment phases of a new World Health Organisation (WHO) WHO-ageism item pool that aims to measure the multi-dimensional nature of ageism. These phases drew on a review of available evidence, an experts’ workshop and structured content validity reviews conducted by experts in scale development and ageism drawn from every world region defined by WHO.

**Results:**

our resulting item pool is designed to provide a multi-dimensional measure of ageism against all ages measured from both a perpetration and experienced perspective and that can produce valid and reliable scores within diverse country contexts and comparable scores across these contexts.

**Conclusions:**

our item pool is the first major step in providing a global and comprehensive measure of ageism. Future phases of research will refine the item pool and establish the statistical psychometric properties of the final tool.

## Key Points

- An instrument that can validly measure the multi-dimensional nature of ageism is currently lacking
- The existence of a reliable and valid scale is critical to advancing efforts to address ageism
- To respond to this need, this study completed the initial phases of development of a new WHO-ageism item pool
- Next stages of development will help reduce and refine this item pool and establish its psychometric properties

## Introduction

Ageism against both older and younger people (defined as aged 50 and over, and younger than 50 years of age, respectively) is prevalent and can be harmful to individual health and wellbeing and to societies at large [1–8]. In 2016 the World Health Organisation (WHO) received a mandate from its member states to lead the Global Campaign to Combat Ageism. An evidence base on ageism was developed, culminating with the first UN Global Report on Ageism [1]. This report highlighted the critical need for an ageism measurement tool that can be used across age groups and countries to help illuminate the scale of ageism, its risk/protective factors and to track the effects of interventions [1]. No existing measures have demonstrated the requisite scope or psychometric properties to meet this need, including recently published measures [9–12]. A key gap is in a measure that can capture ageism against *all* ages [8].

Another gap relates to the need to produce cross-context reliable, valid and measurement invariant scores across a diversity of country settings [13, 9, 14]. This would support valid comparisons across countries and could, for example, facilitate the evaluation of macro-level influences on ageism, such as cultural factors or national policies or legislation designed to tackle ageism [15]. There is evidence for different cultural understandings of ageism and ageism-related concepts such as ‘adolescence’ or ‘older adulthood’ [1] which, if not accounted for, could result in misleading conclusions about cross-country differences.

In view of the limitations of existing scales, the World Health Organisation is pursuing the development and validation of a new self-report scale of ageism that captures all dimensions of ageism and all age groups. The current study describes the initial steps of development of the *WHO-ageism scale*: conceptualisation, item generation and preliminary content validity evaluation. These steps provide the foundation upon which psychometrically robust and content valid scales critically depend. The outcome is a pool of items that will be further tested and validated in the next stages of scale construction, involving data collection from the target populations to assess the statistical psychometric properties of the items (e.g. internal consistency reliability, factorial validity, cross-country measurement invariance and sensitivity to intervention effects).

## Materials and methods

**Figure 1:**
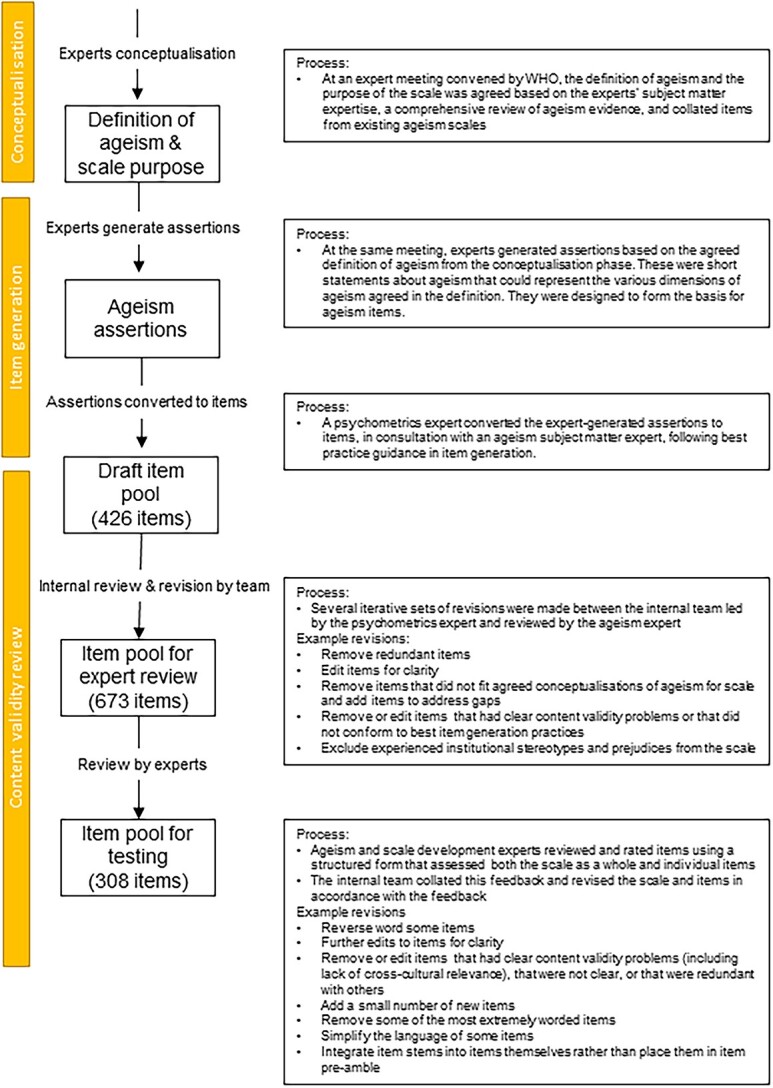
Summary of item review process. Overview of internal and external review and example revisions made at each stage.

This study describes the development of an initial item pool for the *WHO-ageism scale,* which involved: (i) conceptualisation, (ii) item generation and (iii) content validity review (see Figure 1).

### Conceptualisation

Conceptualisation involved defining the concept of ageism that would underpin the scale, as well as its purpose and target population(s). These decisions were based on a comprehensive review of the evidence to understand the nature of ageism, its determinants and manifestations in various settings, as documented in the UN Global Report on Ageism [1] and an international experts’ meeting convened by WHO. The latter involved eight experts (three male, five female) in ageism and/or scale development, with representation from multiple WHO world regions (the African Region (AFRO), the European Region (EURO), the South-East Asia Region (SEARO), and the Western Pacific Region (WPRO)) and age groups.

Experts agreed that ageism encompasses: (i) all age groups (ii) institutional, interpersonal and self-directed forms (iii) experiences and perpetration, (iv) stereotypes, prejudice and discrimination i.e. cognitive, affective and behavioural aspects [1, 16]. A defining feature is that these must be due to the target’s age. For example, being excluded from society *due to age* differentiates the concept of ageism from the closely connected concept of social exclusion. More specifically, social exclusion can be defined as a state in which individuals are unable to participate fully in economic, social, political and cultural life, as well as the process leading to and sustaining this state. Social exclusion can be due to different characteristics (e.g. gender, disability), amongst which is age. Where someone experiences social exclusion as a result of their age, this would represent a form of ageism.

The purpose of the scale was to facilitate globally comparable data on the magnitude, determinants and impact of ageism against young, middle-aged and older individuals and the effects of interventions, with the general population defined as the target population.

Based on this conceptualisation, the experts generated ‘assertions’, i.e. short statements for each aspect of ageism that can provide the basis for items. For example, assertions for *interpersonal experienced discrimination* included ‘my family talks down to me’ and ‘my community isolates me because of my age’.

### Item generation

Item generation built on the expert-generated assertions and was conducted by experts in cross-cultural scale development and ageism. It involved an iterative process of converting assertions to items, with multiple rounds of review and revision and resulted in 426 items. Recommended principles for item generation were followed [17, 18] to minimise possible bias and measurement error, maximise comprehensibility and acceptability and make items as accessible as possible for lower literacy groups. English was the source language and translatability was considered by avoiding idiomatic, metaphorical and colloquial language [19]. The assertions were also reviewed to identify gaps, redundancies or other issues (e.g. a lack of cross-cultural relevance, ambiguity or unclear mapping to the construct definition) and items were modified, added or eliminated accordingly. To support this process, assertions were reviewed against the most recent ageism literature, including that in the reviews documented as part of the UN Global Report on Ageism [1]. These provided a comprehensive state-of-the art overview of contemporary manifestations of ageism. This step led to a revised pool of 673 items.

Refinements regarding the scope of the scale were also made. It was clarified that the focus was on individuals’ perpetration/experiences of ageism as the most direct measures of ageism, rather than on, for example, individuals’ experiences as observers or their perceptions of others’ or broader societal ageist attitudes (e.g. ‘most people in my society think that older adults are a burden’). Similarly, it was clarified that *experienced discrimination* was the only aspect of institutional ageism that could be measured given that institutions do not have cognitions nor affect, and individual respondents cannot be asked about institutional ageism perpetration. A distinction between perpetrated and experienced ageism was not made for self-directed ageism where the respondent plays both roles simultaneously.

Whilst ageism is relevant for children, there are significant challenges in gathering child self-reports [8, 20]. For this reason, we defined target respondents as age 11 and over. This meant that the scale would capture perpetration of ageism against children but not their experienced ageism. Further, based on the idea that respondents could perpetrate different levels of ageism against different age groups and that its manifestation may differ by age [21], separate perpetration items were developed for key age groups. Items were based on the identification of specific manifestations of ageism against different age groups (e.g. stereotypes of adolescents as delinquent versus older adults as a burden) [1]. Finally, in consultation with experts, it was determined that the most suitable model for perpetrated ageism was reflective (reflecting an underlying psychological trait), whereas for experienced ageism it was formative, bringing together experiences in different contexts and in interaction with different individuals [22].

### Other aspects of scale design

#### Administration instructions

Provisional administration instructions were developed that included guidance on obtaining composite scores for subscales, as well as recommendations on capturing additional information alongside ageism scores, including chronological and subjective age, sex/gender, ethnicity and health status [1].

#### Questionnaire introductory sections

Brief introductory texts were drafted for the scale and its sub-sections. In these, neutral language e.g. ‘experiences with other age groups’ was used to avoid the term ‘ageism’ and priming socially desirable responses. Where relevant, pragmatic guidance on age group definitions were provided. *Children* were defined as older than age five but not having gone through puberty. *Adolescence* was defined as puberty/post-puberty but not yet legally an adult. A specific cut-off was not provided for the adolescence/adulthood boundary reflecting variations in legal definitions of adulthood across cultures and *young adulthood* was thus defined as being legally an adult up to around age 35. ‘Around age 35’ was used to provide flexibility to accommodate cross-cultural differences in definitions. *Middle-age* was similarly defined as ‘approximately between age 36 and 64’ and *older adulthood* as ‘approximately aged 65 and above’. Selecting cut-offs that are universally applicable is challenging and these age cut-offs are arbitrary given that conceptualisations of age groups vary substantially across settings according to factors such as life expectancy, and cultural norms around social roles [1, 23]. Further work in the cross-cultural translation and validation of the scale in future phases will examine the possibility of adjusting the age cut-offs and/or labels for each category.

#### Reference period

A specific reference period in psychometric instruments can reduce ambiguity; however, too long a period can increase recall bias and participant burden whereas too short a period risks that important but infrequent manifestations of a construct are missed [18]. Given that some items referenced infrequent events such as being turned down for a job or voluntary opportunity, a 1-year reference frame was selected. A disadvantage of this is that respondents’ retrospective reports may be subject to forgetting and mood-contingent reconstruction.

#### Response format and scoring

A five-point Likert-type scale indicating level of agreement from 1 = *strongly agree* to 5 = *strongly disagree* was selected. Having at least five response options allows greater variation to be captured and increases the likelihood that item scores can be treated as continuous [24]. A ‘don’t know or not applicable’ option was provided to prevent participants from selecting the middle or a random response option when unable to complete the item [25, 26]. Individual item scores were designed to be averaged or used in a latent variable measurement model (as relevant) in the final scale to obtain composite scores.

### Initial content validity review

A sampling frame of experts in scale development and/or ageism was developed based on the ability to review the technical quality of the scale and determine whether items accurately reflected relevant sub-dimensions of ageism, respectively. A panel of 15 experts (eight male, seven female) was recruited, including those involved in the experts’ meeting convened by WHO. For the sub-scales of ageism against children and adolescents, experts in these developmental stages were also sampled. Given the importance of receiving cross-cultural input from early stages of scale development, experts were sampled from every world region defined by WHO [27].

Using a structured form (Appendix 1), experts assessed items for accuracy (whether they correctly reflected the intended constructs, including in their local context), clarity (whether they conform to the principles of good item generation and are likely to be easy to answer for respondents and to translate well into other languages), acceptability (whether they are likely to be acceptable to respondents), and lack of bias (whether they are unlikely to induce systematic bias such as social desirability bias, induce measurement reactivity or are leading). Items were rated as ‘low’, ‘moderate’ or ‘high’ on these criteria, with ‘high’ representing the best rating. Respondents were invited to provide explanations for their ratings and suggestions for improvement, open response comments on the introductory text and response scales, and to comment on any gaps and add suggested items.

All 673 items were assessed by a minimum of four experts (⁓200 items per expert except two experts who were invited to review all items), including at least one expert in scale development and one in ageism, and experts were strategically assigned to review items related to different age groups depending on their expertise on development stage. The assignment of experts to items is summarised in Table 1.

**Table 1:** Summary of items reviewed by each expert

**Items**	**Expert psychometric reviewers (region)** ^**a**^	**Expert ageism reviewers** **(region)**^**a**^
Self-directed ageism (73 items)	12 (AMRO), 13 (EURO), 15 (EURO)	2 (EURO), 5 (AFRO),14 (EMRO)
Experienced ageism (both interpersonal and institutional) (109 items)	7 (EURO), 12 (AMRO), 13 (EURO)	2 (EURO), 3 (WPRO), 6 (SEARO), 10 (AMRO), 14 (EMRO)
Interpersonal perpetrated ageism against children: stereotypes, prejudices, discrimination (80 items)	1 (WPRO), 7 (EURO), 13 (EURO)	2 (EURO), 8 (AMRO), 11 (EURO), 14 (EMRO)
Interpersonal perpetrated ageism against adolescents: stereotypes, prejudices, discrimination (102 items)	1 (WPRO), 13 (EURO)	9 (AFRO), 10 (AMRO), 11 (EURO), 14 (EMRO)
Interpersonal perpetrated ageism against younger adults: stereotypes, prejudices, discrimination (103 items)	13 (EURO), 4 (EURO)	9 (AFRO), 5 (AFRO), 14 (EMRO)
Interpersonal perpetrated ageism against middle-aged adults: stereotypes, prejudices, discrimination (87 items)	13 (EURO), 4 (EURO)	6 (SEARO), 8 (AMRO),14 (EMRO)
Interpersonal perpetrated ageism against older adults: stereotypes, prejudices, discrimination (111 items)	13 (EURO), 15 (EURO)	6 (SEARO), 14 (EMRO)

*^a^* Experts 8, 9 and 14 possessed dual expertise in ageism and scale development but were classified as ageism experts for the purposes of assigning items to reviewers. The items are organised by suggested sub-dimensions; however, future factor analytic work will be required to establish the final optimal dimensions.

### Analysis

Content validity review data was analysed in two stages. First, qualitative feedback was summarised in a hierarchical manner, for each expert and then across experts and subscales, and for the scale as a whole. Scale-level summarised feedback was contested with appropriate justification or implemented at the scale level. Second, item-level qualitative feedback for items that did not achieve a perfect score on all rated dimensions was used as the basis for eliminating or modifying items. As there were many analogous items across subscales, feedback on one item could often be applied to other similar items. Modification was preferred in cases where deletion would result in the loss of a critical aspect of ageism and reduce the representativeness of the scale. Deletion was preferred where a flagged item was redundant with other higher scoring items.

## Results

Scale-level modifications are summarised in Table 2. Item-level deletions, modifications and additions were recorded in a tracking table (available upon request from the corresponding author). An illustrative selection is provided in Appendix 2.

**Table 2:** Scale-level modifications

**Modification**	**Rationale**
Addition of the recommendation to include a measure of intergenerational contact alongside the WHO-ageism scale to the administration instructions	It was highlighted that the perpetrated discrimination responses could be difficult to interpret in the absence of information about the frequency of contact between the respondent and other age groups.
Removal of redundant items	Some items were highlighted as redundant with others, facilitating the removal of one or more items (e.g. ‘middle-aged adults. . .’ ‘. . .lack empathy’ and ‘. . .don’t care about other people’)
Reverse wording of items and/or rewording of items in negative form	All items were initially coded in a negative direction. To help mitigate response biases such as socially desirable or acquiescent responding and to make the scale less negatively framed, some items in each subscale were reversed. Between a third and half of all items were reverse worded. The choice of which items to reverse were based primarily on pragmatic considerations related to which items could easily be made to conform to a simple grammatical structure in reversed form. Reviewers also highlighted items that were worded in negative form e.g. ‘I do not. . .’ as placing a higher burden on participants. These were re-worded to avoid the use of negative forms, in some cases by reversing the wording. (e.g. ‘Others think that because of my age I cannot make decisions for myself’ became ‘Others think that at my age I am able to make decisions for myself’)
Removal of the majority of the most extreme items	Several items were highlighted as extreme/severe by multiple reviewers, with a risk of causing offence or eliciting response biases. The majority of these were removed with a very small number retained to help ensure reliable measurement of ageism at its most extreme levels as well as milder levels. (e.g. ‘I feel disgusted by children’ was removed)
Addition of items covering aspects of ageism not represented in the initial item pool	A small number of items were added based on specific suggestions by reviewers to improve the representativeness of the scale. These covered others assuming a lack of autonomous decision-making capability in a person due to age, making incorrect assumptions due to a persons’ age, and thinking that all people in an age group are alike.
Simplification of the language	Several suggestions for simplifying the language to make it more accessible for younger age groups were implemented. For example, the word ‘finances’ was replaced with ‘money’. Revisions were also made to the pre-amble text to make it more understandable.
Re-structuring the stem-item links	Item stems (e.g. In relation to adolescents, because of their age I think. . .) were originally included as part of the pre-amble text leaving short items in the main body of the questionnaire. An expert in scale translation; however, noted that integrating the stems into the items themselves would improve translatability.
Further exploration of age cut-offs for the pre-amble text	One reviewer suggested that the age cut-off of 65 was too young for older adults. Further stages of scale development will explore different methods of dealing with individual and cultural differences in perceptions of age categories.
Re-word items that relied on a close relationship between the respondent and a person of the target age group	A reviewer noted that some items relied on the respondent having a close relationship with a person of a target age group (e.g. determining their learning choices). To reduce the dependence on this, these items were re-worded to ask about the respondents on whether these behaviours were acceptable rather than whether they engaged in them. (e.g. ‘It is OK for decisions to be made for children without involving them at all’)

In a first stage of item revision based on the expert review, three items were added, 146 substantively modified, 334 eliminated and the remainder unmodified, leaving 342 items. Common reasons for deletion or modification related to: relevance or specificity to ageism, cross-cultural universality, applicability to younger age groups (children and younger adolescents), specificity, comprehensibility, translatability issues, redundancy with other items; and to make the wording less extreme to improve acceptability and/or reduce the risk of socially desirable responding. Examples are provided in Table 3.

**Table 3:** Examples of item level modifications

**Item/text**	**Action**	**Revised item/Text**	**Rationale**	**Revised item/Text step 2**	**Rationale**
Because of my age I. . .generally keep out of things	Remove	–	R12^a^ rated this ‘moderate’ in accuracy and ‘low’ in clarity. It is also redundant with other items capturing the concept of refraining from societal participation. R2 rated this only ‘moderate’ in clarity. R15 noted that this was not clear	–	–
Because of my age. . .there are parts of my neighbourhood, town, or city that are ‘off-limits’	Modify	Because of my age. . .there are parts of my neighbourhood, town, or city where I feel I am unwelcome	R12 noted that ‘off-limits’ is ambiguous. R3 also noted that it is not clear what ‘off-limits’ means. R6 noted that ‘off-limits’ may have translatability issues. R10 noted that this item was not clear in the original wording.	Due to my age, there are parts of my neighbourhood, town, or city where I feel unwelcome	In stage 2, items and stems were integrated
I think that. . . children are a nuisance	Carry forward	I think that. . .Children are a nuisance	No issues were raised with this item by reviewers	Children are a nuisance	In stage 2, items and stems were integrated
In relation to younger adults, I feel. . . A lack of empathy about their problems	Modify	In relation to younger adults, I feel. . . I don’t care about their problems	R13 commented that ‘lack of empathy’ might not be understood by all respondents.	I do not care about younger adults’ problems	In stage 2, items and stems were integrated

*^a^* RX refers to the reviewer number.

In a second stage of revision and in response to expert feedback, stems, which were initially provided as part of the introductory texts, were integrated with items to increase comprehensibility. At this stage the study team eliminated further items based on redundancies and made additional minor improvements for clarity. In a final stage of revision, between one third and one half of items per subscale were reverse worded to mitigate response biases such as acquiescent or socially desirable responding. The final item pool following the revisions includes 308 items.

## Discussion

The purpose of the present study was to develop an initial item pool for the WHO-ageism scale; a scale designed to provide a cross-culturally valid measure of ageism against all ages. Following a rigorous conceptualisation, item generation and initial content validity review process, 308 items were developed. The items are designed to measure stereotypes, prejudices, discrimination in self-directed, interpersonal experienced and interpersonal perpetrated ageism (stereotypes, prejudices and discrimination) against children, adolescents, younger adults, middle-aged adults and older adults, as well as institutional experienced discrimination.

An important challenge, commonly encountered in scale development [13, 28], related to the development of items appropriate for all age groups and across countries and languages. To address this, experts from across the world and with knowledge of different development stages were involved from the earliest stages of scale development. These experts were asked to consider the translatability of items in this initial phase of content validity review and many provided comments on potential translatability difficulties as part of their qualitative feedback. Though it was not feasible for a reviewer from every region to review every item, feedback could be generalised across item sets, which included many analogous items for different targets and from a perpetrated versus experiences perspective. For example, items regarding ‘respect’ were flagged as having potential culture variance by reviewers of some subsets and it could be inferred that the same issue would be applicable to the corresponding items in other sets. Potential issues with translation were amongst the most common reasons for modifying or deleting items and illustrated that transability assessment can be invaluable in addressing issues at an early stage of scale development [29].

In terms of specific items excluded because of universality concerns, one suggested manifestation of ageism in African contexts was accusations of being a witch. However, this was ultimately removed because it was not considered relevant in other contexts. Examples of items that were not included due to their lack of applicability to all age groups included those related to difficulty finding housing and accessing financial products, both of which were considered irrelevant to the youngest age groups. It is possible that further research using, for example, cognitive interviews with target respondents of different ages and living in different contexts may identify further items for modification or deletion to improve the cross- developmental and cultural applicability of the scale.

Future research will focus on gathering further psychometric data for the item pool and validating scales of varying length. This will include a second phase of content validity assessments including both experts and target respondents spanning diverse regional contexts and which gathers and statistically analyses quantitative as well as qualitative content validity data. This will be followed by cross-national data collections to gather data on the test–retest reliability, structural properties and internal consistency reliability, convergent validity, cross-group measurement invariance (e.g. across gender, culture, age) and sensitivity to intervention effects [30]. A child version of the scale will also be considered to address the specific challenges of self-reports by children (e.g. using simplified language and presentation and interviewer-assisted data collection) alongside adapting the content for children (i.e. referencing the specific manifestations of ageism as they occur for children). Similarly, interviewer-assisted and audio versions will be considered for low literacy groups.

Future studies will also help gain wider stakeholder and target population input on the content validity and acceptability of the items, enable testing in a diversity of languages and contexts, and the potential development and validation of additional measures that capture aspects of ageism that may be important but beyond the scope of the WHO ageism pool (e.g. ageism in particular settings such as long-term care institutions). They could also consider the use of measurement methods such as ecological momentary assessment to capture ageism experiences as they happen (as has been done for other concepts), to help overcome the limitations of retrospective questionnaires and to capture its shorter-term variation and influences [31].

## Conclusions

The conceptualisation, item generation and initial content validity review phases of the WHO-ageism scale development suggested that ageism can be measured in a manner that is applicable across age groups and cultures. The output of this phase is an item pool measuring dimensions of ageism against multiple age groups from an experiences and perpetration perspective. The next phase will refine this item pool and establish its psychometric properties.

## Supplementary Material

aa-23-0440-File003

## Data Availability

Data provided in the article and additional data available through the supplementary file provided.
