# Supplementary material for: Development of the item pool for the ‘WHO-ageism scale’: conceptualisation, item generation and content validity assessment
Source: Age Ageing. 2023 Oct 30;52(Suppl 4):iv149–57. doi: 10.1093/ageing/afad105 (PMC10615060; doi:10.1093/ageing/afad105)
Supplement: aa-23-0440-File003 [file aa-23-0440-file003.pdf]

World Health Organization: Measurements of Healthy Ageing.

Development of the item pool for the 'WHO-ageism scale': Conceptualisation, item generation, and content validity assessment

SUPPLEMENTARY DATA

Appendix 1 – Expert Rating form

**The following statements are designed to capture your experiences of interacting with different age groups. Use the response scale provided to indicate the extent to which you agree with the statements below. When answering, think about whether the statement applies in relation to your experiences in the last 12 months.**	**Instructions for experts:**<br><br>**The items that have been assigned to you for review are highlighted in yellow. The other items have been provided for context. We only ask you to review the highlighted items.**<br><br>**Please select 'Don't know', 'Low', 'Moderate', or 'High' from the drop-down menu in each of the columns below to indicate the extent to which you feel the item has accuracy, clarity, acceptability, and carries a low risk of bias.**				
**This section concerns what you think, feel, and do in relation to yourself. Because of my age I think …**	**Accuracy**<br>Does the item measure what it is supposed to measure? Is this true for all cultures you are familiar with? If any of these are a concern the item should be rated 'low' or 'moderate' on accuracy.	**Clarity**<br>Is it clear what the item is asking? Will a respondent be able to easily answer it? Will it translate well into other languages you are familiar with? If any of these are a concern, the item should be rated 'low' or 'moderate' on clarity.	**Acceptability**<br>Is this item likely to cause offense to respondents or otherwise make them feel uncomfortable? If there are concerns about this, the item should be rated 'low' or 'moderate' on acceptability.	**Risk of eliciting bias**<br>Is it likely that this item will create attitudes that did not already exist or elicit socially desirable responding whereby participants give a socially acceptable answer rather than an answer that is a true reflection of their position? If there are concerns that the item may elicit biased responding, it should be rated 'low' or 'moderate' on lack of bias.	**Explanation/further comments**<br>Please use this column to provide any additional explanation of any ratings, to raise any issues with an item not covered by the rating scale, and/or to suggest an alternative item wording. You do not need to complete this for every item, only where you feel supplementary information is helpful to explain a low score or make suggestions to resolve an issue.
**Self-stereotype**					
The following items are designed to measure cognitive aspects of ageis					
I should not ask for money					
I cannot manage my finances					
I have no purpose					

Appendix 2 – Illustrative sample selection of item pool

	Strongly agree	Agree	Neither agree nor disagree	Disagree	Strongly Disagree	Don't know or not applicable
**Self-stereotype**						
The following items are designed to measure cognitive aspects of ageism (what people 'think') in reference to oneself						
At my age, my life has plenty of purpose	O	O	O	O	O	O
I have undesirable characteristics (e.g., selfishness, weakness) as a result of my age	O	O	O	O	O	O
At my age, I can contribute meaningfully to society	O	O	O	O	O	O
**Self-prejudice**						
The following items are designed to measure affective aspects of ageism (what people 'feel') in reference to oneself						
I am embarrassed of my age	O	O	O	O	O	O
I am comfortable with my age	O	O	O	O	O	O
I am proud of my age	O	O	O	O	O	O
**Self-discrimination**						
The following items are designed to measure behavioural aspects of ageism (what people 'do') in reference to oneself						
Due to my age, I limit my participation in decision-making processes (e.g., in my family; at school, college, or work; or in my neighbourhood)	O	O	O	O	O	O
I only wear clothes that I consider age-appropriate	O	O	O	O	O	O
At my age, I express my needs to my family	O	O	O	O	O	O

Interpersonal experienced stereotype

The following items are designed to measure cognitive aspects of ageism (what people 'think') as the respondent experiences ageism directed at them by other people

Others think that my age does not affect my ability to manage my money	O	O	O	O	O	O
Others think that I have nothing valuable to contribute to society because of my age	O	O	O	O	O	O
Others think that I am capable of easily getting along with people of other ages	O	O	O	O	O	O

Interpersonal experienced prejudice

The following items are designed to measure affective aspects of ageism (what people 'feel') as the respondent experiences ageism directed at them by other people

Others admire me because of my age	O	O	O	O	O	O
Others feel anger towards me because of my age	O	O	O	O	O	O
Others feel frustrated with me due to my age	O	O	O	O	O	O

Interpersonal experienced discrimination

The following items are designed to measure behavioural aspects of ageism (what people 'do') as the respondent experiences ageism directed at them by other people

Due to my age, other people talk to me as if I need things simplified	O	O	O	O	O	O
Due to my age, others control my access to healthcare services	O	O	O	O	O	O
Others make decisions for me because of my age	O	O	O	O	O	O

Institutional experienced discrimination

The following items are designed to measure the extent to which the respondent experiences institutional ageism, for example, because of laws and policies that create biases based on age

I have been denied healthcare by a healthcare provider as a result of my age	O	O	O	O	O	O
Policy making process involve people my age	O	O	O	O	O	O
I am treated worse by the police than people of other ages	O	O	O	O	O	O

Interpersonal perpetrated stereotype (against children)

The following items are designed to measure cognitive aspects of ageism (what people 'think') and refer to how the respondent thinks about other people

Children should be involved in the most important decisions	O	O	O	O	O	O
Children are able to understand how the world works	O	O	O	O	O	O
Children are worth listening to	O	O	O	O	O	O

Interpersonal perpetrated prejudice (against children)

The following items are designed to measure affective aspects of ageism (what people 'feel') and refer to how the respondent feels about other people

I like children	O	O	O	O	O	O
Children make me feel sad	O	O	O	O	O	O
I feel comfortable around children	O	O	O	O	O	O

Interpersonal perpetrated discrimination (against children)

The following items are designed to measure behavioural aspects of ageism (what people 'do') and refer to how the respondent behaves towards other people

Decisions should not be made for children without involving them at all	O	O	O	O	O	O
I talk to children as if they need things very simplified	O	O	O	O	O	O
It is OK for adults to control aspects of children's lives that they are capable of managing themselves	O	O	O	O	O	O

Interpersonal perpetrated stereotype (against adolescents)

The following items are designed to measure cognitive aspects of ageism (what people 'think') and refer to how the respondent thinks about other people

Adolescents act without thinking	O	O	O	O	O	O
Adolescents are too young to understand how the world works	O	O	O	O	O	O
Adolescents are responsible	O	O	O	O	O	O

Interpersonal perpetrated prejudice (against adolescents)

The following items are designed to measure affective aspects of ageism (what people 'feel') and refer to how the respondent feels about other people

I distrust adolescents	O	O	O	O	O	O
I find adolescents annoying	O	O	O	O	O	O
I feel frustrated by adolescents' weaknesses	O	O	O	O	O	O

Interpersonal perpetrated discrimination (against adolescents)

The following items are designed to measure behavioural aspects of ageism (what people 'do') and refer to how the respondent behaves towards other people

I talk to adolescents as if they need things simplified	O	O	O	O	O	O
I treat adolescents as if they are ignorant	O	O	O	O	O	O
I take the preferences of adolescents into account	O	O	O	O	O	O

Interpersonal perpetrated stereotype (against younger adults)

The following items are designed to measure cognitive aspects of ageism (what people 'think') and refer to how the respondent thinks about other people

Younger adults can manage their money	O	O	O	O	O	O
Younger adults lack useful knowledge	O	O	O	O	O	O
Younger adults are valuable in the workplace	O	O	O	O	O	O

Interpersonal perpetrated prejudice (against younger adults)

The following items are designed to measure affective aspects of ageism (what people 'feel') and refer to how the respondent thinks about other people

I distrust younger adults	O	O	O	O	O	O
I feel uncomfortable around younger adults	O	O	O	O	O	O
Younger adults annoy me	O	O	O	O	O	O

Interpersonal perpetrated discrimination (against younger adults)

The following items are designed to measure behavioural aspects of ageism (what people 'do') and refer to how the respondent behaves towards other people

It is OK to make decisions for younger adults	O	O	O	O	O	O

I make fun of younger adults	O	O	O	O	O	O
I talk to younger adults as if they need things simplified	O	O	O	O	O	O

Interpersonal perpetrated stereotype (against middle-aged adults)
The following items are designed to measure cognitive aspects of ageism (what people 'think') and refer to how the respondent thinks about other people

Middle-aged adults hold too much power in society	O	O	O	O	O	O
Middle-aged adults get too big a share of society's resources	O	O	O	O	O	O
Middle-aged adults think they know best	O	O	O	O	O	O

Interpersonal perpetrated prejudice (against middle-aged adults)
The following items are designed to measure affective aspects of ageism (what people 'feel') and refer to how the respondent thinks about other people

I feel uncomfortable around middle-aged adults	O	O	O	O	O	O
I find middle-aged adults frustrating	O	O	O	O	O	O
I like middle-aged adults	O	O	O	O	O	O

Interpersonal perpetrated discrimination (against middle-aged adults)
The following items are designed to measure behavioural aspects of ageism (what people 'do') and refer to how the respondent behaves towards other people

I make fun of middle-aged adults	O	O	O	O	O	O
I take middle-aged adults seriously	O	O	O	O	O	O
I take the views of middle-aged adults into account	O	O	O	O	O	O

Interpersonal perpetrated stereotype (against older adults)
The following items are designed to measure cognitive aspects of ageism (what people 'think') and refer to how the respondent thinks about other people

Older adults lack purpose in life	O	O	O	O	O	O
Older adults can manage their money	O	O	O	O	O	O
Older adults are too old to do paid work	O	O	O	O	O	O

Interpersonal perpetrated prejudice (against older adults)

The following items are designed to measure affective aspects of ageism (what people 'feel') and refer to how the respondent thinks about other people

I distrust older adults	O	O	O	O	O	O
I feel comfortable around older adults	O	O	O	O	O	O
I feel frustrated with older adults	O	O	O	O	O	O

Interpersonal perpetrated discrimination (against older adults)

The following items are designed to measure behavioural aspects of ageism (what people 'do') and refer to how the respondent behaves towards other people

I make fun of older adults	O	O	O	O	O	O
I talk to older adults as if they need things simplified	O	O	O	O	O	O
I take over tasks from older adults, irrespective of their capability	O	O	O	O	O	O
